# Effectiveness of Nursing Interventions on Stress and Functional Capacity Among Patients After Coronary Artery Bypass Grafting (CABG): A Randomized Controlled Trial

**DOI:** 10.7759/cureus.116298

**Published:** 2026-09-15

**Authors:** Neena Vir Singh, Raman Kalia, Amarjeet Singh, Sandhya Ghai, Shyam K Thingnam

**Affiliations:** 1 Nursing, National Institute of Nursing Education, Postgraduate Institute of Medical Education and Research (PGIMER), Chandigarh, IND; 2 Nursing, Saraswati Nursing Institute (Baba Farid University of Health Sciences, Roopnagar, IND; 3 Community Medicine, School of Public Health, Postgraduate Institute of Medical Education and Research (PGIMER), Chandigarh, IND; 4 Cardiothoracic Surgery, Advanced Cardiac Centre, Postgraduate Institute of Medical Education and Research (PGIMER), Chandigarh, IND

**Keywords:** cabg, dass-21, duke activity status index, functional capacity, nurse-led interventions

## Abstract

Background: Patients undergoing coronary artery bypass grafting (CABG) frequently experience psychological stress and reduced functional capacity during the postoperative period. This randomized controlled trial assessed the effectiveness of nurse-led interventions on stress and functional capacity in post-CABG patients.

Methods: A prospective parallel-group randomized controlled trial was conducted at a tertiary care hospital in North India. Two hundred adult CABG patients were randomized to receive either nurse-led interventions (educational booklet, visual CD, and face-to-face counseling) or routine postoperative care. Stress was assessed using the Depression Anxiety and Stress Scale (DASS-21) stress subscale, and functional capacity using the Duke Activity Status Index (DASI) and metabolic equivalents (METs). Assessments were performed at baseline, one week, one month, and three months. Data were analyzed using Mann-Whitney U test, chi-square test, effect sizes, and linear mixed-effects models.

Results: Baseline stress and functional scores were comparable between the two groups. At one week, one month, and three months, patients in the intervention group demonstrated significantly lower stress, depression, and anxiety scores than those in the control group (p ≤ 0.001). However, at three months, the intervention group demonstrated significantly greater functional capacity than the control group (p < 0.001).

Conclusion: The nurse-led interventions significantly improved psychological outcomes and functional recovery during the postoperative period in the post-CABG patients.

## Introduction

Coronary artery disease (CAD) is a major cause of morbidity and mortality worldwide. According to the World Health Organization, cardiovascular diseases account for approximately 17.9 million deaths each year [1]. In India, more than 30 million people are estimated to be living with CAD. Coronary artery bypass grafting (CABG) is one of the most frequently performed revascularization procedures for patients with multivessel coronary artery disease [2].

Although CABG improves myocardial perfusion and survival, recovery after surgery is often accompanied by physical and psychological challenges. Patients commonly experience postoperative pain, fatigue, sleep disturbances, anxiety, depression, stress, fear of recurrence, and emotional instability [3]. Stress may result from uncertainty regarding recovery, dependence on family members, lifestyle changes, financial concerns, and fear of complications or death. Persistent psychological stress can adversely affect treatment adherence, wound healing, rehabilitation, and long-term cardiovascular outcomes [4].

Functional capacity is also markedly reduced following CABG surgery. Decreased exercise tolerance, muscle weakness, impaired mobility, dyspnea, and physical deconditioning are common during the early postoperative period because of surgical trauma and prolonged immobilization. These limitations affect patients' ability to perform daily activities, return to work, and participate in social and family life. Assessment of functional capacity, particularly using the six-minute walk test, is therefore widely used to evaluate postoperative recovery and rehabilitation outcomes [5].

Cardiac rehabilitation (CR) is an established component of postoperative care for patients undergoing CABG. Comprehensive rehabilitation programs generally include supervised exercise, patient education, lifestyle modification, psychological counseling, and risk-factor management. Participation in these programs has been associated with improvements in exercise capacity, psychological well-being, cardiovascular risk profiles, and quality of life [6,7]. However, enrollment and adherence remain suboptimal, especially in low- and middle-income countries, because of limited availability of rehabilitation services, transportation difficulties, financial constraints, lack of awareness, and inadequate follow-up after hospital discharge [3].

Nurses are central to postoperative cardiac care and are well positioned to provide education, counseling, symptom monitoring, emotional support, and guidance regarding lifestyle modification throughout the recovery period. Nurse-led interventions have been developed to support self-management, improve adherence to treatment recommendations, encourage physical activity, and promote psychosocial adjustment after CABG surgery [3]. The nurse-led intervention in the present study focused on patient education for managing postoperative stress, anxiety, and depression and emphasized yoga, meditation, breathing and limb exercises, progressive walking, lifestyle modification, and psychological counseling. The walking schedule was structured on a weekly basis, beginning with a 5-minute walk, six times a day, during the first week after discharge. The yoga component included sternal-protective breathing and pranayama practices, including deep breathing, muscle relaxation, guided imagery, *Anulom Vilom pranayama*, *Shavasana*, and *Yoga Nidra*.

Previous studies have reported favorable outcomes of nurse-led rehabilitation strategies. Højskov et al. [8] found that psychoeducational rehabilitation improved both physical recovery and psychological well-being after CABG surgery. Technology-assisted nurse-led follow-up has similarly been shown to facilitate patient engagement and improve postoperative recovery [9]. Akhlaghi et al. [4] demonstrated that structured nursing interventions significantly reduced stress among patients undergoing CABG. Home-based and tele-rehabilitation programs have also been reported to improve quality of life while reducing anxiety and depression after surgery [10]. Moghadam et al. evaluated combined aerobic and strengthening exercise protocols during Phase III CR, with participants training five days a week for five weeks [11]. Those receiving combined aerobic and strengthening exercises showed significantly greater improvements in functional capacity and muscle strength than those receiving aerobic exercise alone (p < 0.05), and these gains were maintained at the three-month follow-up. Fakharirad et al. conducted a quasi-experimental study to evaluate the effect of long-term combined CR and yoga training on heart rate variability among post-CABG patients [12].

Despite these findings, evidence from randomized controlled trials evaluating the combined effect of structured nurse-led interventions, such as supportive educational interventions [13] and audiovisual media-based nursing information [14], on both psychological stress and functional capacity remains limited in India. Given the growing burden of cardiovascular disease and the need for accessible rehabilitation services, evaluating nurse-led interventions may provide evidence to strengthen routine postoperative care and improve patient outcomes.

Therefore, this randomized controlled trial was conducted to evaluate the effectiveness of a structured nurse-led intervention on stress and functional capacity among patients undergoing CABG surgery.

## Materials and methods

Study design and participants

This study was a parallel-group randomized controlled trial (CTRI/2018/02/011897) conducted in follow-up outpatient departments (OPDs) of a tertiary care center in North India from October 2017 to 2021. Ethical clearance (INT/IEC/2017/843, dated September 21, 2017) was obtained from the PGIMER Institutional Ethics Committee. Participants included adults admitted for CABG surgery who were expected to attend the scheduled postoperative follow-up visits and who met the following inclusion criteria: age ≥18 years; intact limb function; ability to read and understand Hindi, English, or Punjabi; and willingness to provide informed consent. Participants who expressed an inability to attend scheduled follow-up visits and those with cognitive impairment or mental disabilities that could hinder participation were excluded. Recruitment took place at the time of admission to the ward before surgery. Baseline assessments of stress, anxiety, depression, and functional capacity were performed after obtaining informed consent. Randomization and allocation to the control and intervention groups were conducted after surgery but before discharge. Participants were informed about the confidentiality of their information and were assured that their decision to withdraw from the study would not affect their routine medical care.

Sample size estimation

The sample size was calculated based on the primary outcome, i.e., the reduction in the stress score (Depression Anxiety and Stress Scale-21, DASS-21) between the intervention and control groups, using the formula for the comparison of two independent means, with the following assumptions: expected standardized effect size (Cohen's d) = 0.50 (moderate effect); level of significance (α) = 0.05 (two-sided); statistical power (1 − β) = 90%; standard normal deviate corresponding to α = 1.96; and standard normal deviate corresponding to β = 1.28.

The sample size was calculated using the following formula:

$n=\frac{2\times \left( Z&alpha;/2 + Z&beta; \right)^{2}}{d^{2}}$

where "n" is the required sample size per group, "Zα/2" is the standard normal deviate corresponding to the significance level (1.96), "Zβ" is the standard normal deviate corresponding to the desired statistical power (1.28), and "d" is the standardized effect size (Cohen’s d) (0.50).

Substituting the values:

$n=\frac{2\times \left( 1.96+1.28 \right)^{2}}{\left( 0.50 \right)^{2}}$

$n= \frac{2 \times \left( 3.24 \right)^{2}}{0.25}$

$n=\frac{2\times 10.4976}{0.25}$

$n=\frac{20.9952}{0.25}$

*n=83.9808*

Therefore, the minimum required sample size was 84 participants per group, resulting in a total sample size of 168 participants.

Considering an anticipated attrition rate of 15%, the adjusted sample size required a minimum of 97 participants per group. To ensure adequate statistical power, account for potential losses during follow-up, and facilitate equal allocation between the study groups, 100 participants were enrolled in each group, resulting in a final sample size of 200 participants.

Participants were recruited using random block sampling and equally allocated to either the experimental group (n = 100), which received the nurse-led intervention face-to-face, or the control group (n = 100), which received routine care only.

Randomization and masking

Eligible patients were enrolled consecutively and then allocated to the study groups using block randomization with random number tables. The block size was two, with two possible allocation sequences (AB and BA). Allocation was performed by an independent faculty member who was not involved in the study to ensure allocation concealment.

All randomized participants were included in the final analysis. As no participants were lost to follow-up and complete outcome data were available for all enrolled participants, the analysis was consistent with the intention-to-treat principle.

Intervention

The nurse-led CR intervention was a structured, nurse-led educational and counseling program. It was developed through a multistep process that included an assessment of existing rehabilitation practices; an extensive literature review; expert validation using the Delphi technique with members from the departments of cardiothoracic surgery, physiotherapy, nursing, and dietetics; followed by pilot testing and reliability assessment. The final program was prepared in the form of a printed booklet (ISBN 978-93-90758-14-2) and an accompanying visual educational CD, both available in English, Hindi, and Punjabi. Participants in the intervention group received this structured CR program in addition to routine postoperative care. The intervention was primarily delivered by a trained nurse, reflecting the pivotal role of nurses in patient education, counseling, and continuity of care after cardiac surgery.

The intervention was delivered through the distribution and explanation of the CR booklet, which emphasized breathing exercises, meditation, yoga, walking, and stress management, together with face-to-face counseling sessions reinforced by visual demonstrations through an educational CD. These sessions were conducted by the investigator during follow-up visits at one week, one month, and three months post-discharge. Each session lasted approximately 30 minutes and focused on reinforcing the educational content, addressing patient concerns, promoting adherence to rehabilitation recommendations, and providing psychosocial support. Participants underwent exercise training five days per week for five weeks, with a gradual weekly increase in intensity. The exercise program included breathing exercises (deep breathing and *Anulom Vilom*, starting at 10 repetitions and progressing to at least 20 repetitions), gentle limb movements, yoga (*Bhramari* and *Yoga Nidra*), and meditation practiced four times a day. The exercise schedule was individualized and progressed according to each participant's capacity and tolerance. Ongoing coordination with the treating physicians and multidisciplinary team, including the physiotherapist and dietitian, was maintained at each follow-up visit, ensuring that the nurse-led CR program complemented routine medical and surgical management without altering prescribed treatment protocols.

The control group received routine postoperative follow-up care and standard instructions provided by the treating physicians, without structured CR education or scheduled counseling sessions.

Data collection tools

Sociodemographic characteristics (age and gender) and clinical characteristics (diagnosis and type of surgery) were obtained using a questionnaire administered at recruitment, before randomization. The DASS-21 [15] was used to assess depression, anxiety, and stress. Specifically, the stress subscale consists of seven items, and the subscale scores were multiplied by 2 to obtain full-scale equivalents, in accordance with standardized scoring guidelines. The Duke Activity Status Index (DASI) [16], a validated self-reported instrument, was used to estimate functional capacity in metabolic equivalents (METs). MET is a unit of functional capacity and not an independent variable. Functional capacity in METs was calculated as METs = estimated VO₂ ÷ 3.5, with estimated VO₂ derived from the DASI. The DASI score was calculated as the sum of "Yes" responses, and peak VO₂ was calculated using the formula: peak VO₂ = (0.43 × DASI) + 9.6.

Data collection method

Recruitment was conducted at admission to the ward for surgery, and baseline assessments of stress, anxiety, depression, and functional capacity were performed after obtaining informed consent. Randomization and allocation to the control (n = 100) and intervention (n = 100) groups were performed after surgery but before discharge. Participants in the intervention group received the nurse-led CR intervention, which focused on stress management and exercise training to reduce stress and improve functional capacity after CABG. Post-intervention assessments were conducted in the follow-up OPD, in consultation with the treating physician to avoid interference with routine care. Investigators performing the assessments were blinded to group allocation to the extent possible in an OPD-based study.

The follow-up schedule included outcome assessments at four time points for stress: baseline (at admission for surgery), follow-up 1 (one week after discharge), follow-up 2 (one month after surgery), and follow-up 3 (three months after surgery). The DASI and METs were assessed at three time points: baseline, one month following surgery, and three months following surgery. Telephone reminders were provided one day before each scheduled patient visit. Because of the COVID-19 pandemic, if a participant missed a scheduled visit, a telephonic assessment was attempted, and the OPD follow-up was rescheduled.

Each session with a participant lasted approximately 30 minutes and included data collection regarding stress-reduction techniques (yoga and meditation) and respiratory and limb exercises aimed at improving functional capacity, with need-based reinforcement provided.

Statistical analysis

Data were analyzed using IBM SPSS Statistics for Windows, Version 22.0 (Released 2013; IBM Corp., Armonk, NY, USA). Descriptive statistics were used to summarize the sociodemographic and clinical characteristics of the participants. Continuous variables were expressed as mean ± standard deviation (SD) or median (interquartile range), as appropriate, while categorical variables were presented as frequencies and percentages. Baseline characteristics between the intervention and control groups were compared using the chi-square test or Fisher's exact test for categorical variables and the Mann-Whitney U test for continuous variables when the assumptions of normality were not satisfied. The primary study outcomes included stress, anxiety, and depression, assessed using DASS-21 scores, as well as functional capacity measured using the DASI and METs. These outcomes were analyzed as continuous variables. To evaluate longitudinal changes over time and account for the correlation among repeated measurements within participants, mixed-effects models with the Kenward-Roger approximation were employed. The models included fixed effects for group (intervention vs. control), time (baseline and follow-up assessments), and the group × time interaction. The interaction term was used to assess whether changes over time differed significantly between the study groups. When significant interaction effects were observed, post hoc between-group comparisons at individual follow-up time points were performed using Bonferroni-adjusted p-values to account for multiple comparisons and reduce the risk of Type I error. For descriptive clinical interpretation, DASS-21 scores were additionally categorized according to standard severity classifications (normal, mild, moderate, severe, and extremely severe). Comparisons of categorical severity distributions between groups were performed using the chi-square test or Fisher's exact test, and effect sizes were estimated using Cramer's V. Effect sizes for continuous outcomes were calculated using r-values derived from the Mann-Whitney U statistic, while effect sizes for categorical outcomes were expressed as Cramer's V. All statistical tests were two-tailed, and a p-value < 0.05 was considered statistically significant.

## Results

A total of 200 patients undergoing CABG were analyzed, with 100 participants each in the control and intervention groups (Figure 1). The sociodemographic and clinical characteristics of the participants are summarized in Table 1. The mean age was comparable between the control group (55.36 ± 5.83 years) and the intervention group (56.98 ± 9.19 years) (t = 1.49, p = 0.140). The control group comprised a significantly higher proportion of male participants (86%) than the intervention group (70%) (p < 0.05). With respect to diagnosis, CAD alone was more frequent in the control group (89%) than in the intervention group (81%), whereas CAD with valvular disorder was correspondingly more common in the intervention group (19.0% vs. 11.0%) (χ² = 2.51, df = 1; p = 0.113). The distribution of surgery type (CABG only vs. CABG with mitral valve replacement) was comparable between the groups (χ² = 3.27, df = 1; p = 0.071).

**Figure 1:**
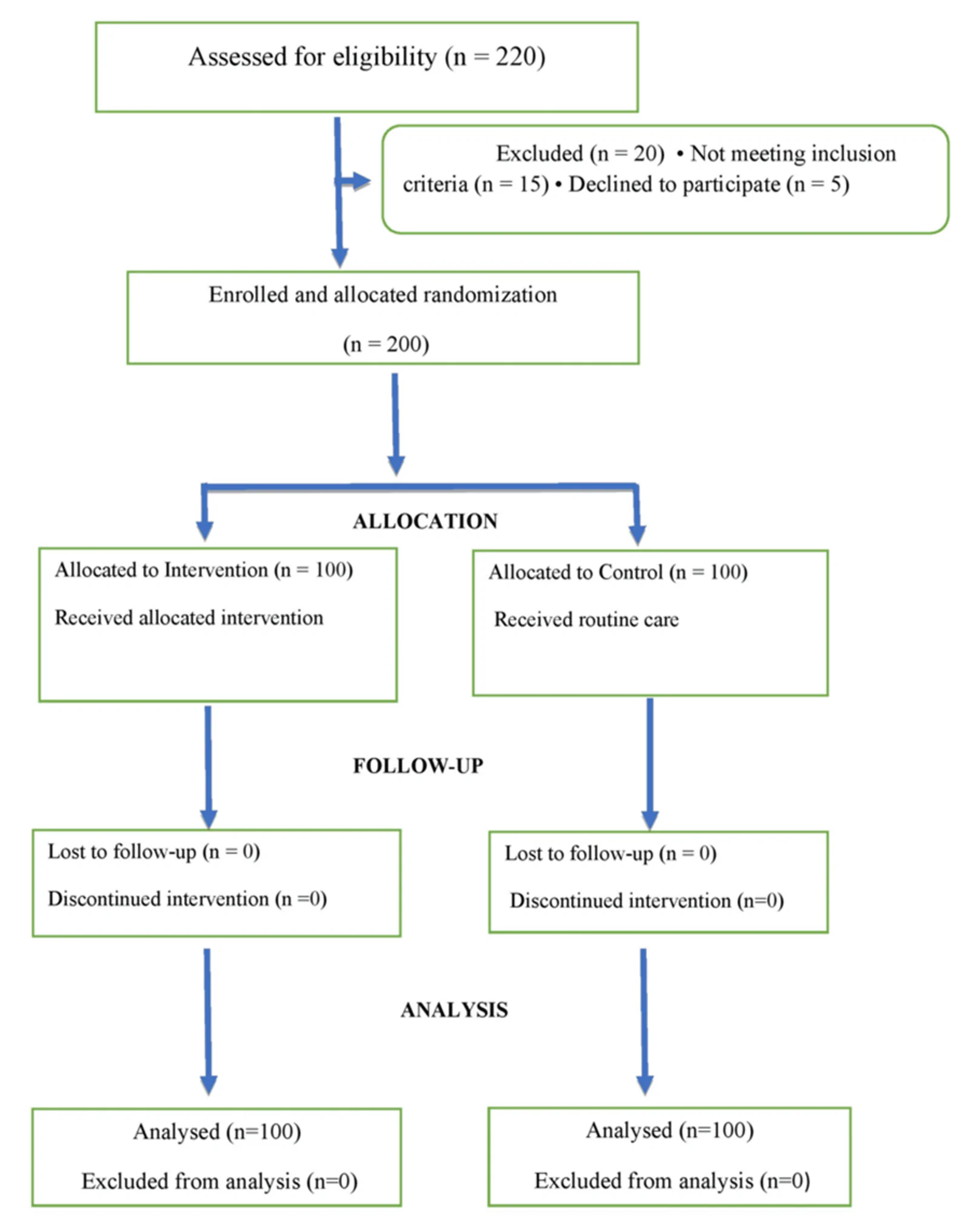
CONSORT diagram of the participants CONSORT: Consolidated Standards of Reporting Trials.

**Table 1:** Sociodemographic and clinical profile of the participants in the control and intervention groups (N = 200)

Variables	Groups		Independent t-test χ²/Fisher (df), p-value
	Control (n = 100)	Intervention (n = 100)	
Age (years), mean ± SD (range)	55.36 ± 5.83(37-68)	56.98 ± 9.19 (37-70)	1.49 (198); 0.140
≤45	10 (10.0)	11 (11.0)	6.53 (3), 0.096
46-55	40 (40.0)	38 (38.0)	
56-65	40 (40.0)	29 (29.0)	
≥66	10 (10.0)	22 (22.0)	
Gender			
Male	86 (86.0)	70 (70.0)	7.459 (1), 0.006*
Female	14 (14.0)	30 (30.0)	
Diagnosis			
CAD only	89 (89.0)	81 (81.0)	2.51 (1), 0.113
CAD with valvular disorder	11 (11.0)	19 (19.0)	
Surgery type			
CABG only	90 (90.0)	81 (81.0)	3.27 (1), 0.071
CABG + MVR	10 (10.0)	19 (19.0)	

Figure 2 shows the longitudinal comparison of DASS-21 stress, depression, and anxiety scores between the intervention and control groups across follow-up assessments. Regarding stress, both groups started with a mean stress score of 14.50 (Figure 2A). At FU1, stress scores increased in both groups, with a greater increase in the control group than in the intervention group. Thereafter, stress scores declined more rapidly in the intervention group. By FU2, the mean stress score had decreased to 18.80 ± 3.87 in the intervention group, compared with 23.20 ± 2.83 in the control group. By FU3, the intervention group had reached a markedly lower mean stress score of 4.18, compared with 10.10 in the control group. regarding depression, both groups began with comparable mean depression scores of 15.80 (Figure 2B). By FU1, the mean depression score had increased sharply in the control group to 33.10 ± 2.63, while the intervention group had a lower mean score of 28.50 ± 2.81. Depression scores subsequently decreased in both groups, but at different rates, with mean scores of 20.40 and 16.80 at FU2 in the control and intervention groups, respectively. The difference between the groups continued to widen. By FU3, the mean depression score had decreased to 4.58 in the intervention group, while the control group had a mean score of 10.18. Regarding anxiety, both groups started with a mean anxiety score of 16.00 (Figure 2C). At FU1, anxiety scores increased in both groups, with a greater increase in the control group (33.40 ± 3.26) than in the intervention group (29.70 ± 2.51). Recovery was more rapid in the intervention group, with the mean anxiety score decreasing to 18.80 ± 4.28 by FU2, compared with 22.50 ± 3.88 in the control group. This difference persisted through FU3, when the mean anxiety score was 9.34 in the control group and 4.92 in the intervention group.

**Figure 2:**
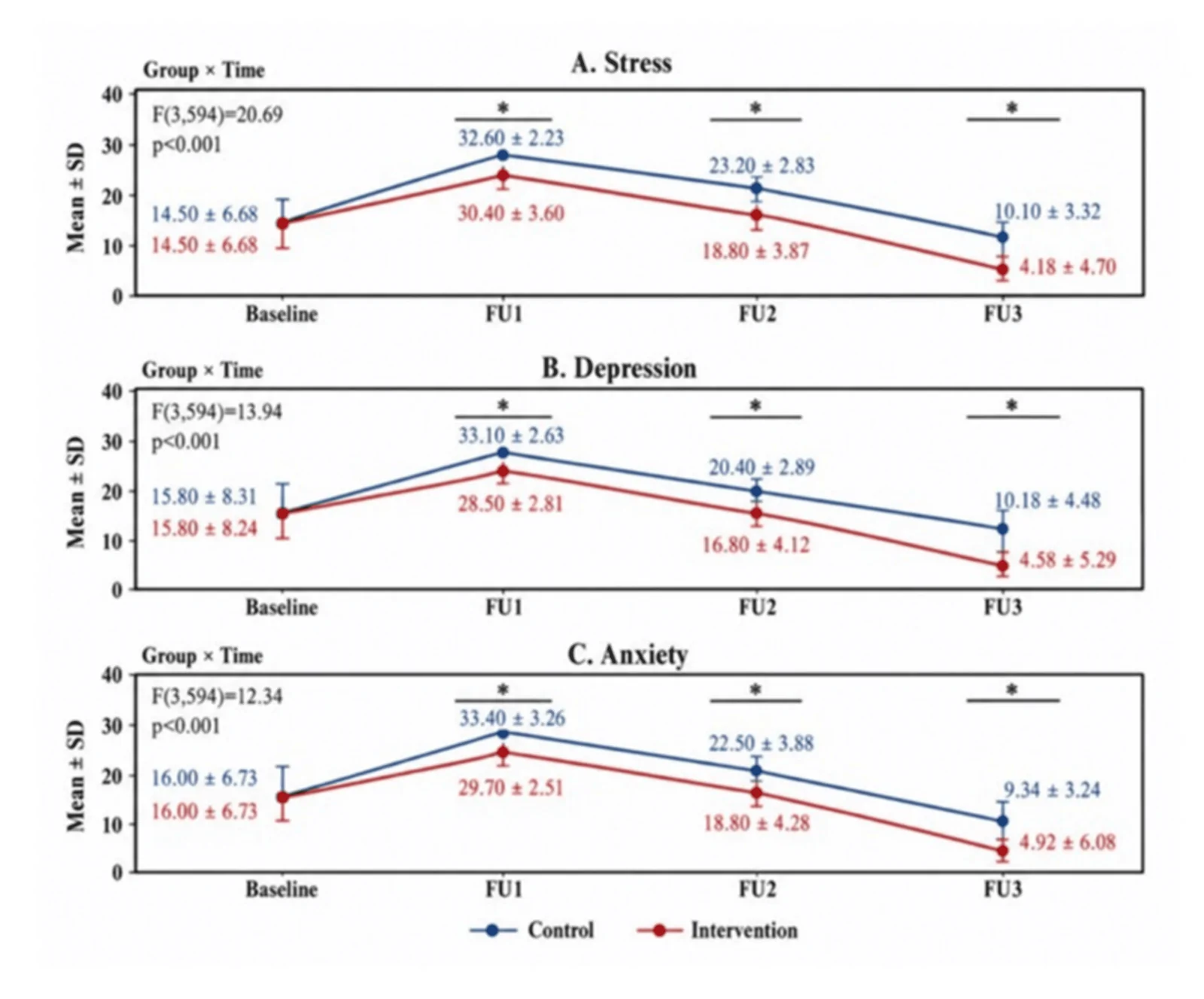
Longitudinal comparison of DASS-21 stress, depression, and anxiety scores between the intervention and control groups across follow-up assessments Values are presented as mean ± standard deviation (SD). Error bars represent ±SD. Asterisks (*) indicate statistically significant between-group differences at the respective follow-up time points (Bonferroni-adjusted p < 0.05). The Group × Time interaction was evaluated using a linear mixed-effects model (LMM). DASS-21: Depression Anxiety and Stress Scale-21.

Functional capacity outcomes, assessed using the DASI and METs, are presented in Table 2. The linear mixed-effects model demonstrated a significant Time × Group interaction for both DASI total (F(2, 495) = 23.72, p < 0.001) and METs (F(2, 495) = 23.72, p < 0.001), assessed at three time points, indicating that the trajectory of functional recovery differed significantly between the two groups. At baseline, there were no statistically significant differences between the control and intervention groups in either DASI or METs, indicating that the groups were comparable at the beginning of the study. At one month, no significant between-group differences were observed in DASI or METs. However, at three months, the intervention group demonstrated significantly better functional capacity than the control group. The mean DASI score was 23.65 ± 10.80 in the intervention group compared with 16.69 ± 6.49 in the control group, with a mean difference of -6.96 (Bonferroni-adjusted p < 0.001). Similarly, the intervention group showed significantly higher METs at three months (5.65 ± 1.33) compared with the control group (4.79 ± 0.80), with a mean difference of -0.86 (Bonferroni-adjusted p < 0.001). The linear mixed-effects model demonstrated a statistically significant Time × Group interaction for both DASI and METs (F(2, 495) = 23.724, p < 0.001). This indicates that the pattern of change in functional capacity over time differed significantly between the two groups, with the intervention group demonstrating greater improvement over the three-month follow-up period compared with the control group.

**Table 2:** Linear mixed-effects model (LMM) with Bonferroni-adjusted between-group comparisons of functional capacity outcomes

Outcomes	Time	Control (C), mean ± SD	Intervention (I), mean ± SD	Mean difference (C-I)	Bonferroni-adjusted p-value	Time × Group interaction		
						F	(df)	p
DASI	Baseline	11.89 ± 4.31	12.48 ± 4.58	-0.59	0.347	23.72	(2,495)	<0.001*
	1 month	11.72 ± 4.15	11.79 ± 2.66	-0.07	0.887			
	3 months	16.69 ± 6.49	23.65 ±10.80	-6.96	<0.001*			
METs	Baseline	4.20 ± 0.53	4.28 ± 0.56	-0.08	0.347	23.72	(2,495)	<0.001*
	1 Month	4.18 ± 0.51	4.19 ± 0.33	-0.01	0.887			
	3 Month	4.79 ± 0.80	5.65 ± 1.33	-0.86	<0.001*			

## Discussion

This randomized controlled trial evaluated the effect of a structured, nurse-led CR program on functional capacity in patients undergoing CABG. The two study groups were comparable in age and type of surgery at baseline; however, statistically significant differences were observed in age-category distribution, gender, and diagnostic profile. The intervention group included a higher proportion of older patients (≥66 years), female patients, and patients with concomitant valvular disorders. These baseline imbalances are acknowledged because they may influence recovery trajectories, as older age, female sex, and concomitant valve surgery are recognized factors associated with slower postoperative functional recovery. Their greater representation in the intervention group suggests that any functional benefit attributable to the intervention was achieved despite a somewhat higher-risk case mix.

The prognostic relevance of these baseline characteristics is well documented in the CABG literature. Krey et al. reported that female patients undergoing combined CABG and valve surgery had significantly higher short-term mortality than men [17]. Female patients had higher 30- and 180-day mortality rates after combined CABG and aortic valve replacement (AVR) surgery, particularly among older age groups, and were generally older and had a greater burden of comorbidities [17]. In a large population-based cohort, Dalén et al. similarly found that women undergoing CABG had a greater burden of cardiovascular risk factors than men, although female sex itself was not an independent predictor of mortality after adjustment for this clustering of risk factors [18]. Consistent with these findings, Isorni et al. observed that women with CAD were significantly older and had a higher cardiovascular risk profile than men at presentation [19]. The overrepresentation of older patients, female patients, and those with valvular disorders in the intervention arm of the present study should therefore be considered when interpreting the functional outcomes.

Functional capacity, assessed using the DASI and DASI-derived METs, showed a significant Time × Group interaction for both measures (DASI: F(2, 396) = 23.66, p < 0.001; METs: F(2, 396) = 24.85, p < 0.001), confirming that functional recovery followed different trajectories in the two groups. At baseline, the groups had comparable scores. At one month, the control group had significantly higher DASI and METs scores than the intervention group. By three months, DASI scores in the two groups had converged and were statistically comparable, indicating that functional recovery had equalized over the study period.

The transient reduction in self-reported functional capacity observed in the intervention group at one month merits careful interpretation, given the higher proportion of older and female participants in this group. Rather than necessarily reflecting poorer physical recovery, this early difference may plausibly be explained by the effect of structured education and counseling on patients' awareness of their own activity limitations. Participants who received detailed guidance on graded activity, symptom monitoring, and avoidance of premature exertion during the vulnerable early postoperative period may have reported their functional status more conservatively and realistically, whereas participants in the control group, who did not receive such structured counseling, may have overestimated their activity tolerance. Importantly, this early difference had resolved by three months, when DASI scores were comparable between the groups, indicating that the intervention did not compromise functional recovery and ultimately supported safe functional recovery.

These functional capacity findings are consistent with the wider evidence base on structured rehabilitation after CABG. In a single-blind randomized controlled trial, Kumar et al. reported that an e-media-supported, exercise-based Phase II CR program produced a significantly greater improvement in functional capacity, measured by the six-minute walk test, than routine care at the 12-week follow-up, together with improvements in quality of life and physical activity levels [20]. Similarly, Miah et al., in a recent systematic review of randomized controlled trials, found that nurse-led CR was broadly comparable or superior to routine care in improving functional capacity and quality of life across diverse cardiac populations, while offering a pragmatic and cost-effective model of delivery [21]. This is further supported by Dwiputra et al. [22], whose randomized trial of a resistance- and aerobic-training CR program in post-CABG patients demonstrated significant gains in functional capacity, including an improvement of 28.2 m in six-minute walk distance and 0.9 METs in aerobic capacity, alongside improved well-being.

Our results extend this literature to a resource-limited Indian setting, demonstrating that a low-cost, nurse-led, booklet- and CD-based program achieves functional recovery comparable to routine care by three months.

Limitations

As this study was conducted during the COVID-19 pandemic, a small number of assessments were performed telephonically. Additional limitations include the reliance on self-reported DASI/METs without objective exercise testing and the single-center setting.

## Conclusions

Incorporating structured, evidence-based nursing interventions into postoperative cardiac care may enhance patients' psychosocial well-being and functional recovery. These findings support the integration of such interventions into routine clinical practice for post-CABG patients to optimize postoperative recovery and improve clinical outcomes.
